# Changes in zebrafish (*Danio rerio*) lens crystallin content during development

**Published:** 2013-02-18

**Authors:** Phillip Wages, Joseph Horwitz, Linlin Ding, Rebecca W. Corbin, Mason Posner

**Affiliations:** 1 Department of Biology, Ashland University, Ashland, OH; 2 Jules Stein Eye Institute, UCLA School of Medicine, Los Angeles, CA; 3 Department of Chemistry, Ashland University, Ashland, OH

## Abstract

**Purpose:**

The roles that crystallin proteins play during lens development are not well understood. Similarities in the adult crystallin composition of mammalian and zebrafish lenses have made the latter a valuable model for examining lens function. In this study, we describe the changing zebrafish lens proteome during development to identify ontogenetic shifts in crystallin expression that may provide insights into age-specific functions.

**Methods:**

Two-dimensional gel electrophoresis and size exclusion chromatography were used to characterize the lens crystallin content of 4.5-day to 27-month-old zebrafish. Protein spots were identified with mass spectrometry and comparisons with previously published proteomic maps, and quantified with densitometry. Constituents of size exclusion chromatography elution peaks were identified with sodium dodecyl sulfate–polyacrylamide gel electrophoresis.

**Results:**

Zebrafish lens crystallins were expressed in three ontogenetic patterns, with some crystallins produced at relatively constant levels throughout development, others expressed primarily before 10 weeks of age (βB1-, βA1-, and γN2-crystallins), and a third group primarily after 10 weeks (α-, βB3-, and γS-crystallins). Alpha-crystallins comprised less than 1% of total lens protein in 4.5-day lenses and increased to less than 7% in adult lenses. The developmental period between 6 weeks and 4 months contained the most dramatic shifts in lens crystallin expression.

**Conclusions:**

These data provide the first two-dimensional gel electrophoresis maps of the developing zebrafish lens, with quantification of changing crystallin abundance and visualization of post-translational modification. Results suggest that some crystallins may play stage specific roles during lens development. The low levels of zebrafish lens α-crystallin relative to mammals may be due to the high concentrations of γ-crystallins in this aquatic lens. Similarities with mammalian crystallin expression continue to support the use of the zebrafish as a model for lens crystallin function.

## Introduction

The ocular lens is a unique model for developmental and cellular biology due to the presence of only two cell types, a superficial epithelium and a deeper mass of denucleated fiber cells, in an easily accessible and transparent organ. Abnormalities in lens cellular function and development can lead to cataracts, a leading cause of blindness in humans [1]. A key to understanding lens function and disease is defining the roles of the diverse lens crystallins, a group of proteins expressed at high concentrations to produce the transparency and refractive properties needed to focus light on the retina. The discovery in 1992 that the small heat shock protein α-crystallin functioned as a molecular chaperone by protecting other lens proteins from stress-induced aggregation provided the first evidence that lens crystallins could serve non-refractive roles [2]. It now appears that α-crystallins have diverse functions in maintaining lens transparency, regulating apoptosis, interacting with cytoskeletal proteins, and regulating fiber cell differentiation [3]. Alpha-crystallins are also expressed widely in non-lens tissues [4,5] where the crystallins are involved in the etiology of multiple neurological [6] and muscular [7] diseases, as well as cancer [8]. Much less attention has been paid to the non-refractive roles of the other major families of vertebrate lens proteins, the β- and γ-crystallins [9-11], and little is known about the possible roles of these crystallins in lens development.

The zebrafish has been used extensively to study retinal development and regeneration [12], and more recently has become a model for investigations of lens function and development [13-15]. Studies have provided detailed morphological descriptions of zebrafish lens development [16-18] and its underlying molecular mechanisms [19-23], and mutant strains have been used to investigate lens cell differentiation and cataract development [24-26]. There are strong similarities in lens organogenesis between zebrafish and mammals [17], and putative conservation in fundamental mechanisms of vertebrate lens development suggests that investigations of the zebrafish lens are broadly relevant to other vertebrate species, including humans [13,15]. Although evolutionary conservation in vertebrate crystallin expression and function can make the zebrafish an attractive model organism for lens research [27-32], differences between zebrafish and mammals are also informative as the differences suggest alternate solutions to common problems of lens biology [33]. A comparative analysis of these differences has already been used to identify specific amino acid changes that increase the protective function of α-crystallin, with potential applications in diverse tissue types [34]. Similarly, while the duplication of αB-crystallin [30] and greater diversity of γ-crystallins [28] can pose a challenge to using the zebrafish as a general vertebrate lens model, these additional crystallins can provide novel approaches for studying lens crystallin function.

A small number of studies have identified ontogenetic changes in lens crystallin protein expression, but little is known about the functional consequences of these changes. Measurements of mouse and rat lens messenger ribonucleic acid (mRNA) indicated changing levels of αA-, αB-, βB1-, and γ-crystallin transcripts during lens development [35-37]. A subsequent analysis of mouse lens protein content by two-dimensional gel electrophoresis (2DE) focused primarily on age-related post-translational modifications, but also identified ontogenetic changes in the expression levels of six crystallins, with three increasing and three decreasing during the first 6 weeks of life, suggesting developmental stage-dependent functions for some crystallins [38]. A more recent study used a shotgun proteomics approach to describe developmental changes in zebrafish lens crystallin expression, with β-crystallins the most prevalent crystallin in the larval lens, and α- and γ-crystallin levels increasing with age [39]. This study also used size exclusion chromatography to calculate the proportion of each crystallin family in the lens from 4.5 days to 2.5 years, finding that α-crystallin levels increased to 22% of the total lens protein by 6 weeks of age, substantially more than previously observed [31].

Here we add a novel set of 2DE data on developmental changes in zebrafish lens crystallin expression from 4.5 days after fertilization to 27 months (near the end of the typical zebrafish lifespan). Most significantly, we identified a shift in crystallin expression between 6 weeks and 4 months of age, during which all three α-crystallins, βB3-crystallin, and the γS-crystallins increased dramatically. We also identified an earlier onset of γN2-crystallin compared to γN1-crystallin, early expression of βB1- and βA2-crystallins, and changes in the relative expression of γM-crystallins. These results are generally concordant with the findings of Greiling et al. [39]. However, our 2DE data indicated that α-crystallin abundance increased from less than 1% in 10-day-old zebrafish lenses to only 6% at 11 and 27 months. Complementary size-exclusion chromatography (SEC) experiments indicated that zebrafish α-crystallins elute with β-crystallins, suggesting that SEC will overestimate α-crystallin proportions in lens protein homogenates. We also found that the total proportion of γ-crystallin stayed relatively constant during lens development, and that aging zebrafish lenses did not contain increasing amounts of large molecular weight protein aggregates. Our visual 2DE proteomic maps of zebrafish lens development allow measurements of absolute abundance for each crystallin and the ability to monitor changes in post-translational modification. These data provide additional foundations for further studies of crystallin function in the zebrafish model system.

## Methods

### Lens protein collection

ZDR strain zebrafish were housed in 10 l aquaria maintained at 28 °C with feedings twice a day of flake food and *Artemia*. Fish were anesthetized by placing them in beakers of water sitting on ice as authorized by Ashland University’s Institutional Animal Care and Use Committee. Lenses were removed when fish were no longer responsive. The number of lenses used depended on the age of the fish as follows: six lenses for fish 10 weeks or older, 15 lenses for 6-week-old fish, and 50 lenses for 10-day and 4.5-day-old fish. Pooled lenses were homogenized in either 600 μl, 200 μl, or 100 μl (respectively, depending on age) of sample buffer (8 M urea, 2% CHAPS, 50 mM DTT, 0.2% Bio-Lyte 3/10 ampholyte, and 0.001% bromophenol blue; Bio-Rad, Hercules, CA) to solubilize total protein. Lens homogenates were centrifuged at 15,000 × *g* for 20 min to remove any unsolubilized material, and the protein in the supernatant was quantified using the RC DC Protein Assay with Microfuge Tube Protocol (Bio-Rad). Spectrophotometric measurements were made with a NanoDrop 1000 (Thermo Scientific, Waltham, MA).

### Two-dimensional gel electrophoresis

Twenty micrograms of lens homogenate for fishes 6 weeks or older or 40 µg of lens homogenate for 10- and 4.5-day samples was focused on immobilized pH gradient (IPG) strips (11 cm; pH 3–10 nonlinear and pH 7–10; Bio-Rad). Second dimension separation was performed on 12% sodium dodecyl sulfate–polyacrylamide gel electrophoresis gels (Pierce, Rockford, IL) and then stained with SYPRO Ruby protein gel stain (Invitrogen, Carlsbad, CA) following the manufacturer’s protocol. SYPRO Ruby stain was used as its intensity increases in a linear relationship to protein abundance [40]. Stained gels were photographed using a Kodak 440 CF Imagestation, and the quantity of protein in each spot was determined with densitometry using Kodak 1D Image Analysis software (Kodak, Rochester, NY).

The densitometry of all crystallin spots on gels produced from pH 3–10 nonlinear IPG strips was compared to that for actin to calculate the relative abundance of each crystallin. Actin was used as a standard since it maintained a relatively constant percentage of total protein abundance (0.59±0.07% of the total protein content in analyzed gels; ±standard error, n=12). Densitometry was also used to calculate the percentage of total lens protein found in each major lens crystallin family (α-, β-, and γ-crystallins). Relative abundance and/or percentage of total protein for gels from 10-day-, 6-week-, and 27-month-old samples were quantified in triplicate from three separate lens preparations and electrophoretic separations. The software package Prism was used to calculate standard error across replicates and to plot the data.

Individual spots were identified by comparing gels with previously published zebrafish lens proteomic maps [31]. Spot identifications were also confirmed with matrix-assisted laser desorption/ionization time of flight mass spectrometry (MALDI-TOF MS) analysis. Gels used for spot identity confirmation were stained with Pierce Silver Stain for Mass Spectrometry (Thermo Scientific).

### Matrix-assisted laser desorption/ionization time of flight mass spectrometry analysis

Gel spots stained with Pierce Silver Stain were selected for peptide mass fingerprinting and subsequently destained according to the manufacturer’s procedure (Thermo Scientific). Gel spots were then prepared for digestion with trypsin overnight with a separate reduction and acetylation method. Digested solutions were acidified with 0.1% trifluoroacetic acid and mixed with the matrix alpha-cyano-4-hydroxycinnamic acid. Samples were analyzed with MALDI-TOF mass spectrometry using a Bruker MicroFlex instrument (Bruker Daltonics, Billerica, MA). Externally calibrated, positive-ion mass spectra were obtained in reflection mode. The resulting mass spectra were compared to theoretical peptide mass maps using ProteinProspector software and the NCBI non-redundant protein database.

### Size exclusion chromatography

Lens homogenates from various ages were fractionated with AKTA fast protein liquid chromatography (FPLC; GE Healthcare) on a Superose HR6 column (50 mM NaPO_4_, 0.1 M NaCl, pH 7.0) and the resulting peaks analyzed with sodium dodecyl sulfate–polyacrylamide gel electrophoresis (SDS-PAGE; 12.5% gel) to identify crystallin families.

## Results

**Figure 1:**
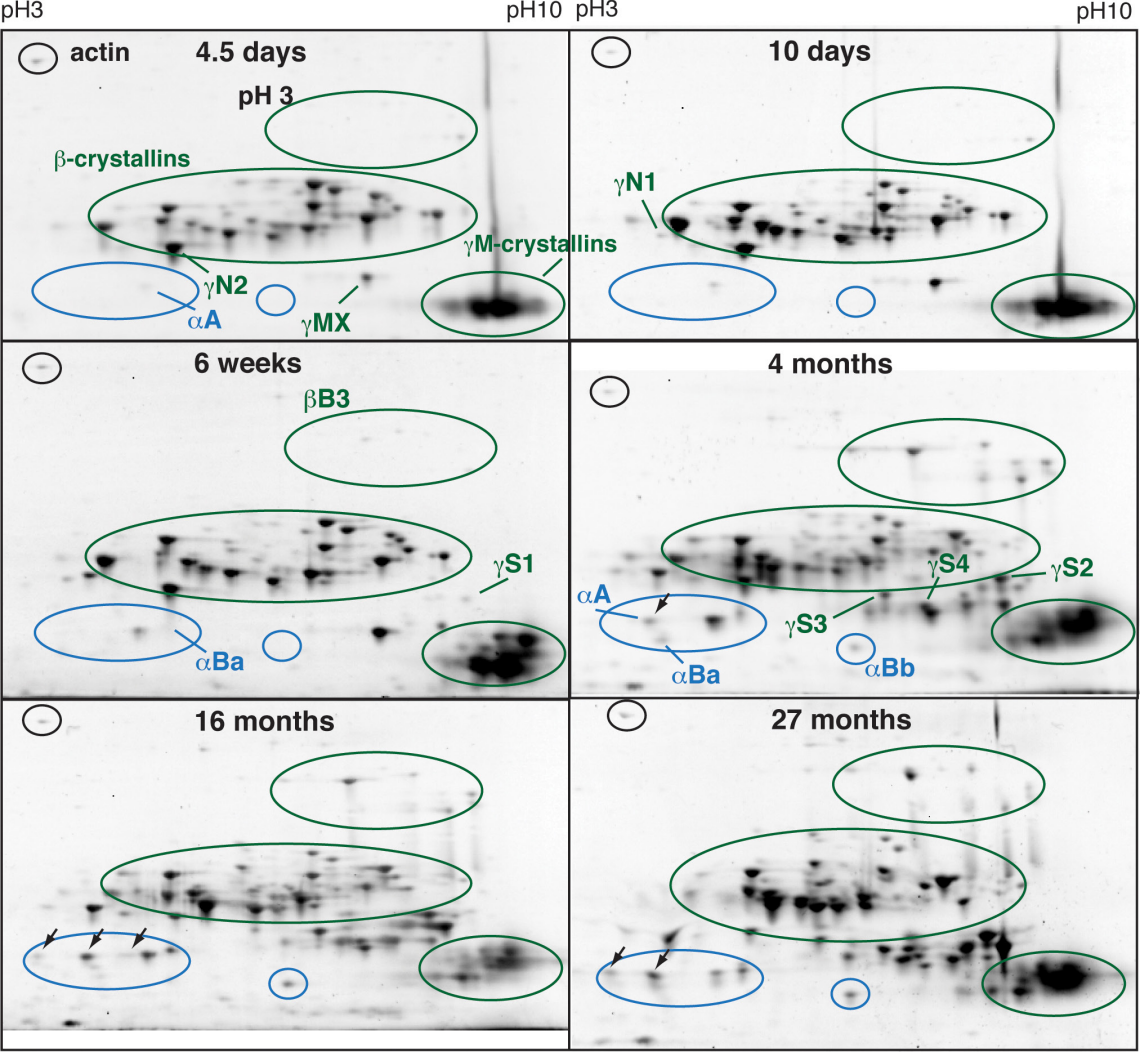
Two-dimensional gel electrophoresis of total zebrafish lens protein shows age-specific expression patterns. Separation was performed on 11 cm pH gradient 3–10 nonlinear immobilized pH gradient strips. Ovals indicate the location of different crystallin groups, and labels note their first appearance. Alpha-crystallins are shown in blue and β/γ-crystallins in green. Black arrows indicate phosphorylated αA-crystallin [31]. Spot identifications relied on previously published proteomics maps [31] and were confirmed with matrix-assisted laser desorption/ionization time of flight (MALDI-TOF) mass spectrometry. Gels were stained with SYPRO Ruby Red.

**Figure 2:**
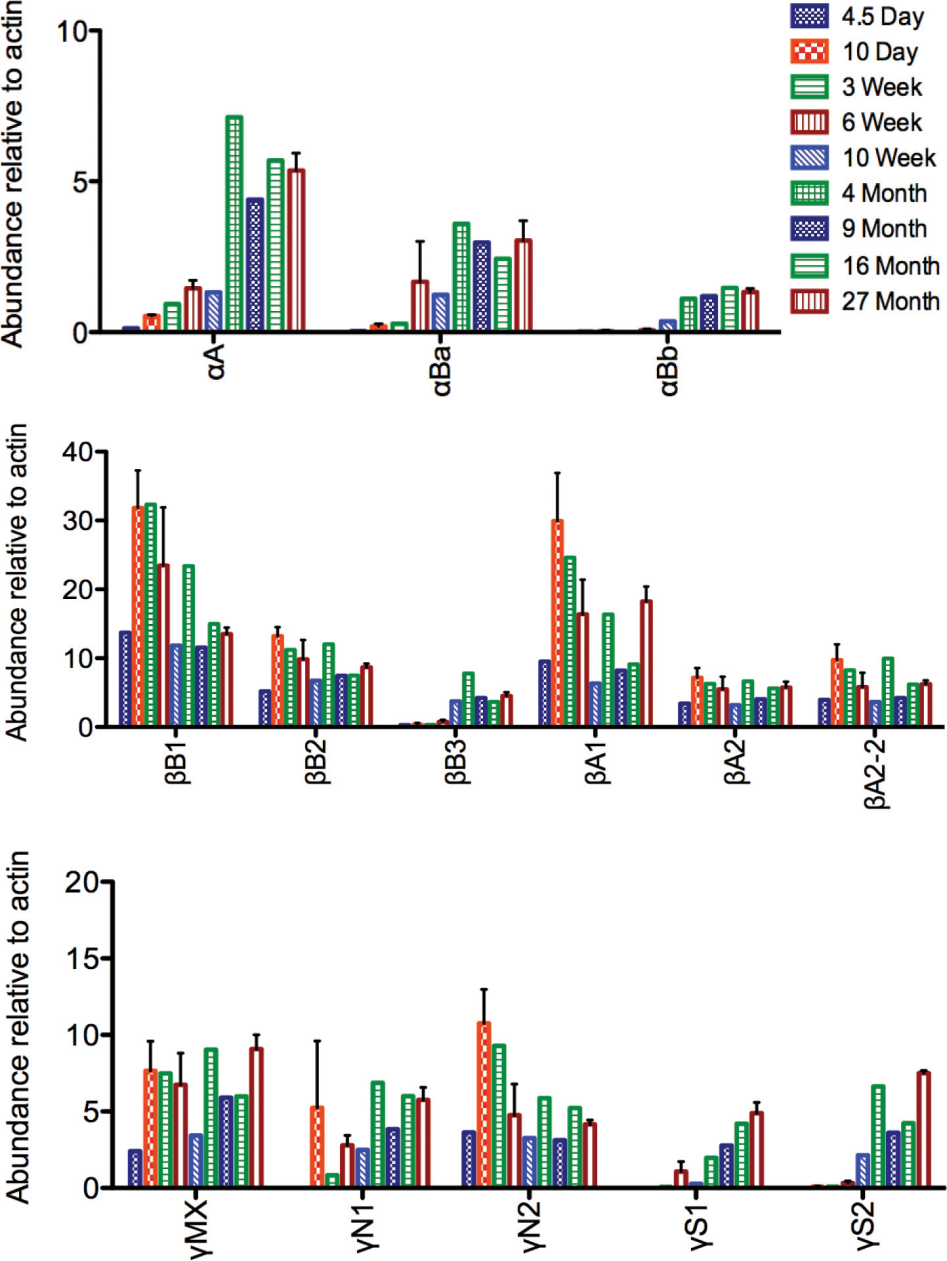
Abundance of select zebrafish lens crystallins at different ages indicates a shift in expression between 6 weeks and 4 months. The proportional intensity of each crystallin spot was quantified by calculating its pixel density relative to actin. Error bars for the 10-day, 6-week, and 27-month time points indicate standard error of the mean (n=3). Multiple crystallins show a marked increase in expression between 6 weeks and 4 months of age.

**Figure 3:**
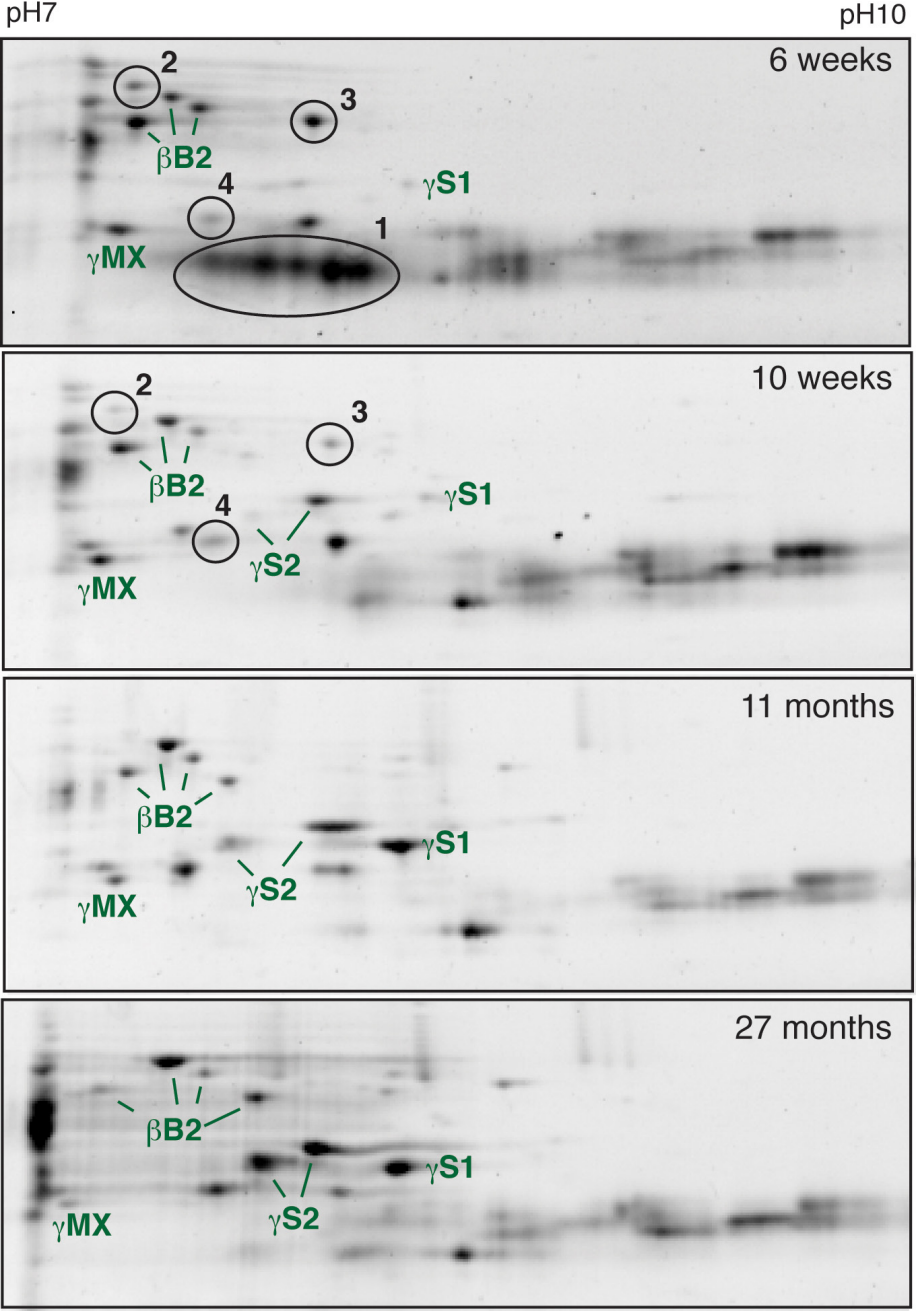
Separation of total zebrafish lens protein using 7–10 pH strips identifies ontogenetic changes in β/γ-crystallin expression. The identities of several crystallins found in each gel are noted. Numbered ovals indicate protein spots found only in the 6- and 10-week lenses.

Two-dimensional gel electrophoresis of total lens protein homogenates from 4.5 day to 27 month post-fertilization zebrafish showed ontogenetic changes in the relative abundance of specific crystallins, with a dramatic shift between 6 weeks and 4 months (Figure 1). Alpha A-crystallin first appeared in the earliest lenses examined (4.5 dpf) and underwent a substantial increase in abundance between 6 weeks and 4 months (Figure 1). Both αBa- and αBb-crystallin first appeared at 6 and 10 weeks, respectively, and showed similar increases after 10 weeks post-fertilization (Figure 1 and Figure 2). Phosphorylated forms of αA-crystallin were noticeable by 4 months and increased with age (Figure 1, black arrows). Beta-crystallins were abundant at all developmental stages with the exception of βB3-crystallin, which was expressed at low levels until 10 weeks of age (Figure 1 and Figure 2). Beta B1- and βA1-crystallins were the most abundant lens proteins through 6 weeks post-fertilization, peaking in expression at 10 days to 3 weeks and then decreasing as the fish aged (Figure 2). Beta B2-, βA2-, and βA2-2-crystallin were found at relatively constant levels throughout development. The developmental timing of γ-crystallin expression varied between subtypes. The aquatic specific γM-crystallins were abundant as early as 4.5 dpf (Figure 1). Gamma N2-crystallin was also prominent at 4.5 dpf, with γN1-crystallin noticeable by 10 dpf and prominent by 6 weeks. In contrast, the four observed γS-crystallins were not noticeable until 6 weeks or later, and increased in abundance with age (Figure 1, Figure 2). Because many of the γM-crystallins cooccur as a large mass of spots when separated on pH 3–10 2DE strips, we also focused lens protein homogenates on higher isoelectric range pH 7–10 strips. Electrophoretic separations resulting from these strips showed a large number of low molecular weight spots at 6 weeks that disappeared by 10 weeks (Figure 3, oval 1). Based on the molecular weight, these likely were γM-crystallins. Three additional proteins occurred in the 6- and 10-week samples but did not appear in adult lenses (Figure 3, ovals 2, 3, and 4). Ovals 2 and 3 are likely β-crystallins based on molecular weight, while oval 4 could be a β-crystallin or γM-crystallin. Insufficient protein was collected from these spots for positive identification with MALDI-TOF mass spectrometry.

**Figure 4:**
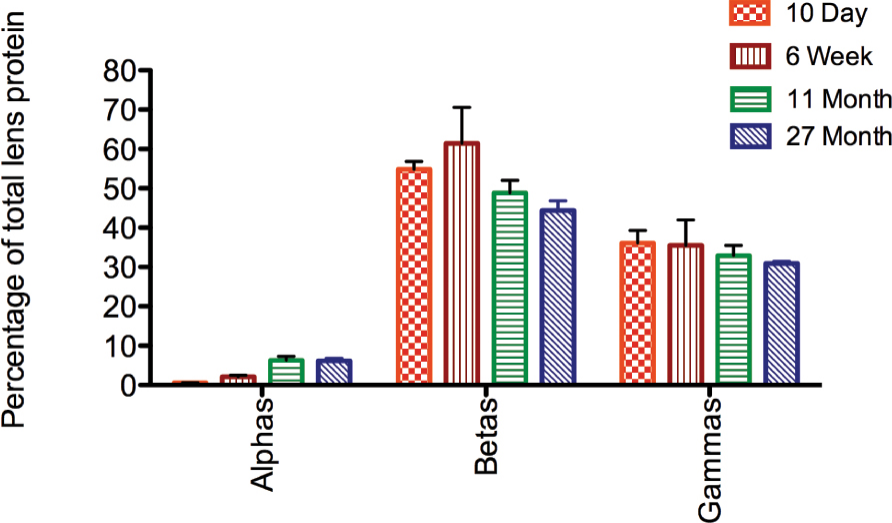
Proportion of α-, β-, and γ-crystallins in the zebrafish lens at different ages. Percentages were calculated by measuring the pixel densities of all spots for each crystallin family and comparing to the total protein content on two-dimensional electrophoresis gels. Error bars indicate standard error of the mean (n=3).

The pixel density of all 2DE spots was used to determine the percentage of total zebrafish lens protein composed of each crystallin family at different developmental stages. Alpha-crystallins grew from less than 1% of total lens protein at 10 dpf to a maximum of 6.3% in the adult lens (Figure 4). The total amounts of β- and γ-crystallins remained relatively constant during development, although, as shown above, the individual components varied (Figure 2). Beta- and γ-crystallins comprised approximately one half and one third of the total lens protein, respectively (Figure 4).

**Figure 5:**
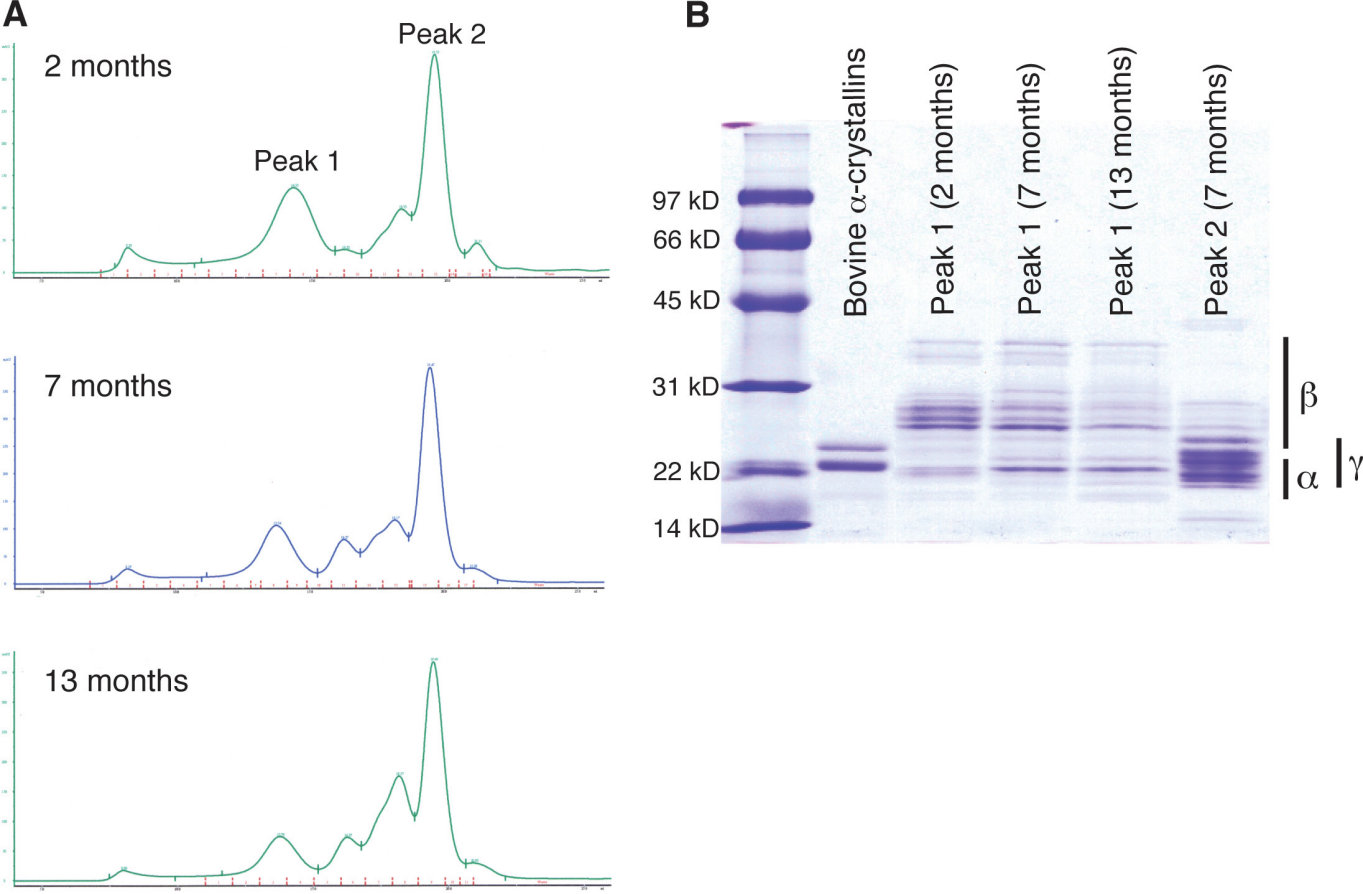
Size exclusion chromatography of zebrafish soluble lens protein at three ages. Resulting chromatography peaks (**A**) were subsequently analyzed with one-dimensional gel electrophoresis and compared to purified bovine α-crystallin (**B**) to determine the purity of the zebrafish crystallins in peak 1. This peak contained a mixture of α- and β-crystallins in all three ages examined, as determined by comparing the band sizes to typical ranges for each crystallin family (noted to the right of the panel). The molecular sizes of the standards used are indicated.

Soluble lens protein from juvenile and adult zebrafish were analyzed with SEC followed by sodium dodecyl sulfate–polyacrylamide gel electrophoresis analysis. SEC produced five peaks in addition to the void volume, similar to profiles produced with mammalian lenses [41,42]. However, unlike mammalian protein the high-molecular-weight peak (Figure 5A, peak 1) contained a mix of α- and β-crystallins (Figure 5B). This α-/β-crystallin peak appeared to elute earlier in the older fishes, suggesting an increase in the relative proportion of higher mass α-crystallins in this mixture with age. The largest peak produced by SEC contained a mixture of β- and γ-crystallins (Figure 5A, peak 2). There were no apparent increases in large molecular weight aggregated protein eluting with the void volume as the lens aged.

## Discussion

Our 2DE examination of the developing zebrafish lens identified three ontogenetic patterns in lens crystallin expression suggestive of stage-specific functions. First, βB1-, βA1-, and γN2-crystallin peaked in expression before 10 weeks post-fertilization (Figure 2). A second group consisting of all three α-crystallins, βB3-crystallin, and the γS-crystallins increased in expression between 6 weeks and 4 months, while a third group containing βB2-, βA2-, βA2-2-, γMX-, and γN1-crystallin was expressed at relatively constant levels throughout development. Chromatographic examination of lens homogenates not only confirmed that the adult zebrafish lens contains lower α-crystallin concentrations than mammalian lenses but also found low levels (<1%) in the larval lens, suggesting that vertebrate lens development may not necessarily require the higher amounts seen in mammals. The lack of large molecular weight protein species in the elution of aged zebrafish lenses likely reflects the functional antiaggregation-chaperoning ability of this species’ α-crystallins.

Comparison of our 2DE data with those from a prior shotgun proteomics study [39] emphasizes the benefits of each approach and their complementary nature. For example, Greiling et al. [39] identified several novel γM-crystallins restricted to larval stages that 2DE can detect (Figure 3, 6 weeks, oval 1) but not directly sequence. Although the shotgun approach can rank-order crystallins by abundance, 2DE adds the ability to measure absolute amounts of each crystallin based on spot densitometry, provides visual maps of the changing lens proteome, and identifies patterns in post-translational modification. The identification of increasing phosphorylation in zebrafish αA-crystallin with age (Figure 1, black arrows), similar to the pattern seen in mammals, provides further evidence that zebrafish α-crystallin is a valid model for investigating mammalian α-crystallin function. The 2DE and shotgun approaches identified similar ontogenetic patterns in protein expression. One exception was seen with βB2-crystallin, which was not identified in 4.5-day lenses by the shotgun approach, but was seen at that stage in 2DE gels (Figure 1). Similarly, shotgun data did not include βB1-crystallin in 6-week lenses, while it was the most abundant protein seen in our 2DE analysis at that stage and was abundant in shotgun-analyzed samples at all other stages. These discrepancies suggest that although shotgun and 2DE approaches can converge on similar results, the former may miss identifying some proteins.

Some of the same ontogenetic shifts in zebrafish lens crystallin expression documented here have also been found in mammalian lenses. The delayed expression of γS-crystallins and early expression with subsequent reduction in βB1-crystallin is shared between the zebrafish and the mouse [38]. Interestingly, mammals and zebrafish contain one β-crystallin that is not expressed until later developmental stages. However, in mammals, this is βB2-crystallin [38], while in zebrafish, it is βB3-crystallin, suggesting that a function specific to later stages of development may be attached to different β-crystallins in mammals and fishes. A similar transfer of functions may also explain why different β-crystallins in mammals and fishes contain C- and N-terminal proline-arginine and proline-asparagine extensions (βB2-crystallin in mammals and birds compared to βB3-crystallin in fishes) [31,32,43]. Shared patterns in mammalian and zebrafish crystallin expression add to a growing set of evidence that the fundamental biology of the vertebrate lens and function of many lens crystallins is evolutionarily conserved [17,28,31]. Although the γM-crystallins are restricted to aquatic vertebrates, the γN- and γS-crystallins are found in all major vertebrate taxa [28], making the zebrafish a possible model for understanding their function. Although the specific function of γN-crystallins is not known, zebrafish γN1- and γN2-crystallin exhibit different degrees of solubility within lens protein homogenates, possibly reflecting divergent interactions with cell membranes or cytoskeleton [31]. The earlier expression of γN2-crystallin relative to γN1-crystallin identified in this study is concordant with a possible divergence in function during lens development (Figure 1).

One significant difference between zebrafish and mammalian lens crystallin expression is the amount of α-crystallin. Although mice express all three of their α-crystallins as newborns, with only αB-crystallin increasing with age [38], the three zebrafish α-crystallins are almost nonexistent in early stage embryos. Zebrafish αA-crystallin expression occurs before that of both αB-crystallins, similar to rats in which αB-crystallin is not expressed until 18 days of fetal development [37]. The zebrafish α-crystallin levels in our 2DE data increased significantly after 10 weeks of age, similar to results from a prior shotgun proteomics study [39], but never attained the levels found in the mammalian lens. Although Greiling et al. [39] found αA-crystallin was the most abundant lens protein in 6-month-old zebrafish, our 2DE data indicate that this crystallin is less abundant or similar in abundance to several individual β- and γ-crystallins. Our estimate of 6.3% and 6.2% total α-crystallin in 11- and 27-month-old adult zebrafish lenses is similar to calculations from previous studies for fishes [31,44] but is significantly lower than a recent measurement using size exclusion chromatography [39], which estimated abundance by calculating the area under the first major peak to elute from a size exclusion chromatography column. Although this peak typically contains pure α-crystallin when eluted from mammalian lens homogenates, previous studies suggested that fish α- and β-crystallins are more tightly associated and will coelute in this peak [44,45]. Our present data confirm that this initial peak is a mixture of α- and β-crystallins, indicating that measurements of this peak area may overestimate α-crystallin abundance.

Lower levels of α-crystallins in zebrafish lenses relative to mammals is not likely due to a loss in the importance of their chaperone-like activity. Zebrafish αA- and αBb-crystallin, as well as multiple other fish αA-crystallins, are strong molecular chaperones, suggesting that antiaggregation protective activity has been maintained during vertebrate evolution [29,30,34]. As in mammals, zebrafish α-crystallins’ ability to prevent the aggregation of stressed or aging proteins likely helps to conserve lens transparency, and explains why we did not see high molecular weight aggregates in our SEC elution profiles (Figure 5). The reduction of chaperone-like activity only in the lens-specific zebrafish αBa-crystallin suggests that chaperone function of αB-crystallins in general may not be necessary in the lens, but is instead tied to roles in extra lenticular tissues. Because of ubiquitous expression, mammalian αB-crystallin is thought to be a multifunctional protein, and retaining chaperone activity in mammalian αB-crystallin could be important for antiaggregation properties in muscle or nervous tissue, but not lens. Zebrafish and mammalian α-crystallins share two other features that suggest conservation of functions. Alpha-crystallins in zebrafish and humans are expressed at similar ratios of about 3:2 αA to αB in the adult lens [31,41], and αA-crystallins in both taxa undergo an age-dependent increase in phosphorylation [41] (Figure 1). The reason for the reduced amount of α-crystallin in the zebrafish lens remains unclear. One possibility is that the large concentrations of γM-crystallins required to produce a high-density aquatic lens may simply exclude greater amounts of α-crystallins. Similarly, the rat lens contains low α-crystallin and high γ-crystallin concentrations relative to other mammalian species [46]. Another unanswered question is why the embryonic zebrafish lens contains such extremely low levels of α-crystallin. The knockout of both α-crystallin genes in mice leads to abnormal embryonic lens development [47], and previous studies suggest that reduced αA-crystallin levels in embryonic zebrafish lenses can lead to cataract or lens regression [25,48]. Therefore, the presence of some amount of zebrafish α-crystallin may be required, but the levels apparently do not need to be as high as in mammals. The roles of specific zebrafish lens crystallins could be examined by blocking their translation with synthetic oligonucleotides [49], although this approach could be complicated by functional redundancies in the large number of β- and γ-crystallins.

This study provides the first developmental series of 2DE proteomic maps for the zebrafish lens and identifies several ontogenetic shifts in lens crystallin expression that suggest stage-specific functions for some proteins. Our 2DE and size exclusion chromatography data also confirm that zebrafish lenses express lower levels of α-crystallins compared to mammalian lenses. The parallels between the ontogenetic expression patterns in mammalian and zebrafish lenses and the ability to efficiently manipulate gene expression in zebrafish using antisense oligonucleotide morpholinos and the introduction of various transgenes makes this species an excellent model for studying the role of crystallins in lens development and function.
